# Positive Design Framework for Carer eSupport: Qualitative Study to Support Informal Caregivers of Patients With Head and Neck Cancer in Sweden

**DOI:** 10.2196/45748

**Published:** 2023-05-30

**Authors:** Awais Ahmad, Shweta Premanandan, Ulrica Langegård, Åsa Cajander, Birgitta Johansson, Maria Carlsson, Ylva Tiblom Ehrsson

**Affiliations:** 1 Division of Visual Information and Interaction Department of Information Technology Uppsala University Uppsala Sweden; 2 Department of Informatics and Media Uppsala University Uppsala Sweden; 3 Genetics and Patology, Experimental and Clinical Oncology Department of Immunology Uppsala University Uppsala Sweden; 4 Lifestyle and Rehabilitation in Long-Term Illness Department of Public Health and Caring Sciences Uppsala University Uppsala Sweden; 5 Otorhinolaryngology and Head and Neck Surgery Department of Surgical Sciences Uppsala University Uppsala Sweden

**Keywords:** eHealth, subjective well-being, positive design, adaptability, informal caregivers, head and neck cancer, preparedness to care

## Abstract

**Background:**

Informal caregivers of patients with head and neck cancer (HNC), such as the patient’s spouse, other close relatives, or friends, can play an important role in home-based treatment and health care. Research shows that informal caregivers are usually unprepared for this responsibility and need support with taking care of patients and other daily life activities. These circumstances place them in a vulnerable position, and their well-being may be compromised. This study is part of our ongoing project Carer eSupport, which aims to develop a web-based intervention to facilitate informal caregivers in the home environment.

**Objective:**

This study aimed to explore the situation and context of informal caregivers of patients with HNC and their needs for designing and developing a web-based intervention (Carer eSupport). In addition, we proposed a novel framework for the development of a web-based intervention aimed at promoting the well-being of informal caregivers.

**Methods:**

Focus groups were conducted with 15 informal caregivers and 13 health care professionals. Both informal caregivers and health care professionals were recruited from 3 university hospitals in Sweden. We adopted a thematic data analysis process to analyze the data.

**Results:**

We investigated informal caregivers’ needs, critical factors for adoption, and desired functionalities of Carer eSupport. A total of 4 major themes, including information, web-based forum, virtual meeting place, and chatbot, emerged and were discussed by informal caregivers and health care professionals for Carer eSupport. However, most study participants did not like the idea of a chatbot for asking questions and retrieving information and expressed their concerns such as a lack of trust in robotic technologies and missing human contact while communicating with chatbots. The results from the focus groups were discussed through the lens of positive design research approaches.

**Conclusions:**

This study provided an in-depth understanding of informal caregivers’ contexts and their preferred functions for a web-based intervention (Carer eSupport). Using the theoretical foundation of designing for well-being and positive design in the informal caregiving context, we proposed a positive design framework to support informal caregivers’ well-being. Our proposed framework might be helpful for human-computer interaction and user experience researchers to design meaningful eHealth interventions with a clear focus on users’ well-being and positive emotions, especially for informal caregivers of patients with HNC.

**International Registered Report Identifier (IRRID):**

RR2-10.1136/bmjopen-2021-057442

## Introduction

### Background

Head and neck cancer (HNC) cases are increasing globally, and the treatment and rehabilitation process requires many resources from medical caregivers [1]. During treatment, patients with HNC may experience functional impairments, such as problems with speaking, saliva, chewing, and swallowing; they may also experience aesthetic, appearance, and social issues [2]. This may have long-lasting effects on patients and their informal caregivers (hereafter referred to as caregivers). Caregivers are spouses or partners, relatives, friends, or neighbors who have a strong personal relationship with the patient and usually provide care to the patient in the home environment [3]. With some external help, education, and training, caregivers can help patients transition from hospital to home environment [1,4,5]. These patients receive varying degrees of extensive treatments in hospitals and often require support from their caregivers at home. Caregivers need to quickly learn and adopt caregiving skills for these severely impaired patients [6]. Hence, caregivers play an important role in helping such patients with home-based treatments and health care; however, this is a challenging task for them. Caregivers are often not well prepared to take up caregiving activities and help patients adjust to new life realities [6-10]. This can compromise the physical and mental health of caregivers. Several studies have also highlighted that caregivers of patients with cancer might experience emotional, social, and physiological issues [1,6,8].

Recent literature has addressed some aspects of people’s subjective well-being when using IT apps [11-15]. However, the needs and situations of caregivers of patients with severe and life-threatening diseases, such as cancer, have not been adequately addressed. Desmet and Pohlmeyer [16] proposed a positive design framework to support human flourishment and users’ subjective well-being. In addition, Peters et al [17] adopted positive psychology guidelines and presented another framework with a special focus on the user’s psychological well-being, in which they stress users’ well-being to be addressed according to their psychological needs, such as autonomy, relatedness, and competence. Gulliksen et al [18] proposed the key principles of user-centered system design, emphasizing the importance of understanding the user’s context and environment to provide better usability. However, their work was solely focused on organizational and professional work environments. The studies described earlier provide a general overview of designing for well-being and support the idea of involving users in the early stages of system design and development. Understanding users’ contexts and preferences is an important aspect of designing for well-being.

### Informal Caregivers’ Burden

Many informal caregivers may not feel fully prepared to take on the responsibilities and challenges that come with providing care, especially if they have not received any training or support [7]. Several factors can contribute to the lack of preparedness of caregivers such as a lack of full understanding of the medical condition or care needs of the person they are caring for. They may also not have access to the necessary resources, such as equipment or supplies, to provide care [19]. This is aggravated by caregivers experiencing a caregiving burden. It is the stress experienced by a caregiver caused by the demands of providing care and balancing it with their personal responsibilities, such as managing their time, maintaining their social roles, managing their financial resources, and maintaining their emotional well-being [19]. Studies have shown that caregivers are more likely to have symptoms of poor physical and mental health or anxiety and depression when compared with noncaregivers [12]. Such situations may also lead to insomnia, reduced well-being, and a decreased willingness to care. In addition, they may also struggle with financial burdens because of their caregiving responsibilities while lacking a support system to help them with their caregiving duties, resulting in feelings of isolation and stress [13]. Many caregivers also have other responsibilities, such as work and family obligations, which make it difficult for them to find time to provide care. Hence, it is vital for caregivers to seek out resources and support to help them feel more prepared and equipped to handle their caregiving responsibilities. This may include finding information and education about the medical condition or care needs of the person they are caring for, seeking financial assistance or respite care to alleviate some of the burden, and connecting with other caregivers for support and advice [8]. Hence, it is important to support them to alleviate their caregiving burden and improve their well-being.

### Informal Caregivers’ Well-being and Positive Design

Human well-being and flourishing are integral elements of any technology [14]. People use and adopt technologies that fulfill their needs to enhance their physical and physiological well-being [15]. Therefore, technology should have a clear impact on users’ well-being and enhance their positive emotions. Designers should understand the user group context and their needs and investigate factors that may enhance their well-being [4]. Involving different stakeholders, especially users, is vital in the design of IT apps [20,21]. However, involving users in the design process is insufficient; designers and developers should deliberately focus on the factors that positively impact users’ well-being and flourishing [4,14,15,22].

The term “positive design” is used as an umbrella term for design approaches and research in which the main intention of the design is the subjective well-being of people and communities [16]. Positive design principles and concepts are derived from positive psychology [21], which discusses concepts and procedures for enhancing human flourishing and subjective well-being [23]. Desmet and Pohlmeyer [16] used positive psychology guidelines for human flourishment to propose a positive design framework for well-being. They emphasized that designers should have an explicit intention to support individuals’ desire to flourish and live pleasurable, enjoyable, and satisfying lives. They established 3 fundamental elements of positive design: design for virtue, design for personal importance, and design for pleasure. They argued that each of these elements should independently stimulate human well-being, whereas the intersection of these elements enables and stimulates human flourishing. Design for virtue refers to the design of products and services that encourage virtuous behavior and support people’s values and goals. This refers to designing products that make it easier for people to engage in activities that align with their personal values, such as exercise or volunteering. Design for personal importance involves designing products and services that help people feel a sense of purpose and meaning in their lives. This refers to designing products that allow people to engage in activities that align with their personal goals and values or that allow them to express their identity and personality. Design for pleasure involves designing products and services that provide enjoyment and pleasure to users. This can involve designing products that are esthetically pleasing or that provide a sense of accomplishment or satisfaction to the user. By considering these strategies in the design process, designers can create products and services that not only function well but also have a positive impact on the well-being and happiness of users.

To address basic psychological needs for well-being, Peters et al [17] explicitly translated the concepts of psychology into the human-computer interaction (HCI) context. They suggest that various factors, if used while designing an IT app, will contribute to positive well-being in users. These factors are autonomy (independence in pursuing one’s goals and moral values), competence (ability and effectiveness), and relatedness (being connected to other related people).

Zhang [15] investigated the fundamental factors that motivate people to adopt and use a given technology. He stressed that people tend to adopt a given technology when they feel it would support their subjective well-being by fulfilling their basic needs in their daily activities. Therefore, technology-enhanced interventions should focus on the users’ quality of life and well-being for better adoption. Zhang [15] highlighted motivational needs such as autonomy, relatedness, competence, and achievement as the basic precursors of successful technology adoption.

The above-described studies give us an overall idea of how positive design concepts can be and should be used to design meaningful technologies that fulfill users’ basic needs, simulate their positive emotions, and ultimately enhance subjective well-being. However, there is still a need to understand how these general principles and guidelines can be practically implemented in a specific context such as informal caregiving. Therefore, in this study, we also discuss caregivers’ preferences for a web-based intervention from a positive design perspective.

### Carer eSupport Project

This project comprises a multidisciplinary research team, including researchers from HCI and software engineers and cancer nursing and medical researchers within the HNC field. The overall goal of Carer eSupport is to prepare caregivers of patients with HNC for caregiving and to decrease their caregiving burden with the help of a web-based intervention called “Carer eSupport.” User needs and preferences for Carer eSupport were gathered from caregivers and health care professionals. On the basis of this, the first version of Carer eSupport will be designed and developed. Thereafter, feasibility studies will be conducted to evaluate the effectiveness and acceptability of the first version, the results of which lead to the second version of Carer eSupport. Finally, the effectiveness, usability, relevance, and acceptability of Carer eSupport will be tested in a randomized controlled trial. Further details about the project can be accessed from our study protocol “Internet-based support for informal caregivers of individuals with head and neck cancer (Carer eSupport): a study protocol for the development and feasibility testing of a complex online intervention” [10].

### Aim

In this study, we explored the context of caregivers of patients with HNC and their needs for a web-based intervention (Carer eSupport) in Sweden. Critical factors that might influence the adoption of such web-based interventions were also discussed. In addition, the study participants also highlighted the desired functionalities and characteristics of Carer eSupport. The findings of this study assist us in answering the following research questions (RQs):

- RQ1: What are the preferred functions of informal caregivers of patients with HNC in designing a web-based intervention to support their well-being?
  RQ1.1: What should be the characteristics of different preferred functions in web-based interventions?
- RQ2: What are the facilitators and barriers to adopting the web-based intervention from the perspectives of informal caregivers of patients with HNC and health care professionals?
- RQ3: How can positive design guidelines support informal caregivers’ well-being in patients with HNC?

This study contributes to research on caregivers in 3 ways. First, major preferences for a web-based intervention (Carer eSupport) are highlighted from the perspectives of caregivers and health care professionals. Second, facilitators and barriers to the adoption and acceptability of web-based interventions in a specific context are emphasized. Finally, general guidelines for “designing for well-being and positive design” in a particular context of caregivers of patients with HNC are proposed, and eventually, a novel framework “Positive Design Framework for Informal Caregivers” to support caregivers is presented.

## Methods

### Design

This study adopted a qualitative research approach. Our ongoing project Carer eSupport [10] was used as a case to address the contextual nature of caregivers’ well-being. In this study, we conducted focus group discussions with different stakeholders. The focus group method is a qualitative research approach for gathering empirical data on a specific topic with focused and well-organized discussions in small groups of carefully selected people [24]. To understand the context of caregivers and their preferences for Carer eSupport, focus groups were conducted with the caregivers. Thereafter, focus groups were also conducted with health care professionals to make Carer eSupport adaptable and acceptable for clinicians for possible future implementation in routine cancer care. All focus groups were conducted using a web-based videoconferencing tool [25].

### Participants and Data Collection

We recruited 15 caregivers from 3 university hospitals in Sweden. A contact person at the oncology and radiotherapy clinics screened individuals with HNC who had an identified caregiver. We enrolled adult participants (aged >18 years) with different stages of HNC who were about to initiate treatment, were undergoing treatment, or had completed treatment within the past 3 months. Thereafter, we contacted each individual with HNC, and if they provided consent, contacted their caregivers to participate in the study. A total of 24 caregivers were invited to participate, and 15 of them consented to participate in the study. Cognitive impairment and inability to understand, speak, or read Swedish were the exclusion criteria for the caregivers.

Thereafter, 25 health care professionals were invited to participate, of which 14 agreed. They were aged approximately 30 years, and the majority of the participants were female. The inclusion criterion for the study was that health care professionals must have prior experience with patients with HNC.

Drawing from the existing literature regarding the needs of caregivers of patients with HNC as well as from health care professionals, 2 interview guides were formulated to conduct focus groups with caregivers and health care professionals (Textboxes 1 and 2). Focus group questions were formulated and developed through a collaborative brainstorming process among the authors UL, YTE, ÅC, and AA. The focus groups addressed questions from the following themes: experiences of being a caregiver, perceptions of IT-based support, support needs of caregivers, and health caregivers’ perceptions of internet-based support.

**The interview guide for informal caregivers.**

**Experiences of being a relative of a person with head and neck cancer**

- How did your life change when your relative was diagnosed with head and neck cancer?
- What are or were your needs as a caregiver of the patient with head and neck cancer?
- What support do or did you receive that met your needs?
- What support do or did you lack in your role as a family member?

**Informal caregivers’ perceptions of an internet-based intervention**

- What are your experiences of using the internet for support?
- Describe your experiences with any IT programs or applications you have used in health care and medical care.
- How do you think that internet-based support for relatives should work?
- Follow-up questions:
  What functions should be available?
  How to communicate with others?
  How should the information be presented?
  How should the application’s layout look like?
  Should it be possible to customize the appearance, and if so, how?
  Will there be a need for IT support?
  How do you think a nonphysical person (robot) answers your questions?

**The interview guide for health care professionals.**

**Support needs of informal caregivers**

- What are the support needs of relatives of people being treated for head and neck cancer?
- Which medical professions are needed to support relatives?
- Is there support other than nursing care that the relative may need?

**Health caregivers’ perceptions of an internet-based intervention**

- How do you think that internet-based support for relatives should work to be usable and useful?
- Follow-up questions:
  What functions should be available?
  How to communicate with others?
  How should the information be presented?
  How should the application’s layout look like?
  Should it be possible to customize the appearance, and if so, how?
  Have you previously used any technology-enhanced solution (eg, video meetings on Skype) to help or contact patients or their relatives? If yes, what challenges did you face?
- How do you think a nonphysical person (chatbot) answers your questions?
- What would be the key factors that may contribute to the long-term utilization of Carer eSupport for several years?

### Data Analysis

To analyze the data, we adopted the 6-step thematic data analysis process by Braun and Clarke [26]. We used both inductive and deductive approaches to analyze the data. Initially, we used an inductive approach to investigate the needs and preferences for Carer eSupport among caregivers of patients with HNC. Subsequently, we used a deductive approach to examine the positive design guidelines in the specific context of caregivers of patient with HNC to support their well-being. The recordings of the focus groups were transcribed and stored in the data analysis software. AA thoroughly and repeatedly read transcripts as the first step of thematic analysis to familiarize himself with the data and inductively explore the initial ideas. The basic ideas related to the study aim were transformed into initial codes in the second step using positive design guidelines for well-being by AA in discussions with ÅC. The third step was to examine the codes and identify broader and more important themes. Study participants’ relevant data, such as quotations and observations, were collected for these broader themes. As the focus groups were conducted in the native language (ie, Swedish), the selected quotations were translated into English. These themes were further reviewed and refined in the fourth step. In this step, we also ensured that all themes were directly or indirectly related to answering the RQ. AA and SP performed steps 3 and 4. The fifth step selected and finalized the most relevant and important themes. A workshop was conducted with all the study authors to thoroughly discuss and finalize the themes. Finally, the major themes, linked quotations, and researchers’ commentaries are presented in the *Results* section.

### Ethics Approval

All research procedures were approved by the Swedish Ethical Review Council (Dnr:2020-04650). Before starting the focus group discussions, the study participants were informed in written form and verbally about their rights and the study’s implications. They were also informed of the purpose and procedures of the project. To ensure the security and integrity of the study participants, an end-to-end encrypted videoconferencing tool was used to conduct the focus groups. All gathered data were stored in a safe and secure database at Uppsala University.

## Results

### Study Participants

A total of 15 caregivers were selected from 3 hospital universities in Sweden. In total, 2 caregivers could not manage the time for the focus group discussions; therefore, they were interviewed individually. The characteristics of the caregivers are presented in Table 1.

**Table 1:** The characteristics of informal caregivers.

Informants	Sex	Age (years)	Relation with patient	IT literacy	Education
IC.1	Female	64	Partner	Medium	University master’s level
IC.2	Male	58	Spouse	High	University >3 years
IC.3	Male	54	Spouse	High	University bachelor’s level
IC.4	Female	34	Daughter	High	University master’s level
IC.5	Female	58	Spouse	High	University master’s level
IC.6	Male	70	Spouse	Medium	Secondary school
IC.7	Female	34	Daughter	Medium	Secondary school
IC.8	Female	64	Ex-wife	High	University master’s level
IC.9	Female	72	Spouse	Medium	University bachelor’s level
IC.10	Female	75	Spouse	Medium	Secondary school
IC.11	Female	55	Spouse	High	Secondary school
IC.12	Female	63	Spouse	Medium	University bachelor’s level
IC.13	Female	46	Daughter	High	University master’s level
IC.14	Male	66	Son	High	University master’s level
IC.15	Male	55	Spouse	Medium	Secondary school

Focus groups were also conducted with health care professionals to ensure that Carer eSupport is adaptable and acceptable for clinicians and can be implemented in routine cancer care. It also helped us to understand the patients’ disabilities and their needs for care from the health care professionals’ viewpoint. In total, 13 health care professionals were recruited from different hospitals in Sweden. All health care professionals were carefully selected from different fields of caregiving to patients with cancer, including nurses, physicians, dietitians, dentists, and speech therapists. The characteristics of health care professionals are presented in Table 2.

**Table 2:** The characteristics of health care professionals.

Informants	Professional role	Professional experience (years)
HCP.1	Nurse	5-10
HCP.2	Nurse	>10
HCP.3	Research assistant, nurse	5-10
HCP.4	Dietitian	>10
HCP.5	Physician	5-10
HCP.6	Hospital almoner	3-4
HCP.7	Specialist nurse	<10
HCP.8	Dental hygienist	5-10
HCP.9	Assistant nurse	<10
HCP.10	Speech therapist	5-10
HCP.11	Physician	5-10
HCP.12	Speech therapist	5-10
HCP.13	Speech therapist	<10

### Findings

#### Overview

**Figure 1:**
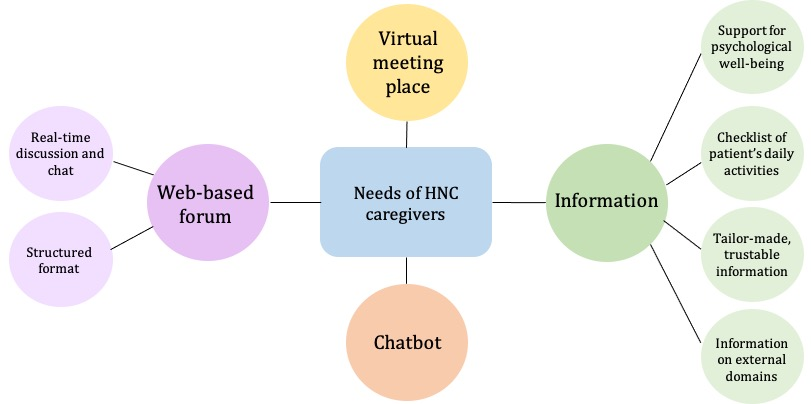
Themes and subthemes identified. HNC: head and neck cancer.

This section presents the findings of our focus group discussions with caregivers and health care professionals. Figure 1 presents the themes and subthemes identified by the focus groups. To support caregivers’ well-being, study participants highlighted their desired functions on the Carer eSupport platform. The study participants also discuss the facilitators and barriers to successful adoption and the specific characteristics of those functions. The following subsections present the participants’ views and citations.

#### Information

Most participants emphasized the need for tailored and trustable information for caregiving, their own psychological and mental well-being, and receiving help for their daily life activities. Both caregivers and health care professionals believed that this information should be available digitally and in a format that is easily understandable to caregivers. This information should be presented in a manner that caregivers from nonmedical backgrounds can understand. Health care professionals also stressed that the information about diagnosis and caregiving needs to be provided in an easy-to-access way by structuring them categorically. Most caregivers spent time finding information from external sources and stressed that access to such information in one place would benefit them by reducing their time and effort they spent. Health care professionals felt that most information available on the web or offline could be too general. Most caregivers in the study felt a sense of virtue, acknowledgment, and competence (based on positive design guidelines) through tailor-made and trustable information functionality.

#### Tailor-made and Trustable Information

Both caregivers and health care professionals suggested that information should be precise according to caregivers’ needs and situation-specific and tailored information. They believe that the information currently available is usually generic about diseases and treatments; however, the same disease may have different side effects for different individuals. Hence, they insisted on having access to tailor-made and trustable information. On the basis of this, it can be noted that the generic nature of information can be a barrier to successfully adopting a web-based intervention:

I think it is difficult with all written information that it very quickly becomes very general and held for a large group where it is only a small, small part that is related to me. And in such a case, I actually become very selfish. I really don’t care how it is for everyone else, but I just want help with something very specific for me.

IC.3, Table 1

Although most participants highlighted the importance of tailored information, some health care professionals showed their concern that specific information would not be easy to provide and could create an extra burden for health care professionals, who considered it as a barrier to practical implementation. To avoid this potential barrier of extra workload on health care professionals, they suggested having some general information. In this way, health care professionals do not have to work extra, but caregivers may obtain some important and relevant information:

I think it might be best to keep the information general as well. For example, the information about the most common operations...as well as the most common side effects or this is how the radiation treatment works but if you would have something specific for each patient, then there is something extra to be imposed on us.

HCP.5, Table 2

#### Support for Caregivers’ Psychological Well-being

Most caregivers highlighted the need for well-being and psychological well-being. They suggested a collection of pages with inspirational and motivational videos and mindfulness exercises to help calm them. They felt that such resources could have a positive effect on their mental health and may help reduce stress. The caregivers also felt that such support could positively affect the quality of care provided to their relatives:

It would be quite nice if you had a small collection of pages, not only regular caregiving but something interesting e.g. a movie on the immune system movie. If you have many thoughts and worries, your sleep will probably be affected, maybe it is good with a mindfulness exercise to sort of calm down and get rid of thoughts...even like small video clips, a story or an interview where you can read about a fictional person who is close relative where he tells about what he has done as a relative. As it can also give inspiration...

IC.13, Table 1

Health care professionals also confirmed the need for information and resources related to caregivers’ health and well-being. They stated that caregivers would like to have acknowledgment and confirmation about what they are going through in their daily life. They also want to be acknowledged for their work on caregiving:

I believe that the need for information and facts also exist to make you feel that you can care for your relative, but then you have your feelings and how I feel as well...When I care for my relative and what needs arise at my side. It feels like two different things, and when you design this support, everything should be included. Therefore, you need to divide it, e.g. these are facts for how I should care for my relative, and then, this is the support for me [as a relative].

HCP.2, Table 2

#### Checklist of Daily Life Activities With a Patient With HNC

All caregivers in our study stressed the need for information regarding their daily life activities. Therefore, a collection of pages containing information about daily life activities as the caregiver of a patient with HNC would be helpful. For example, a checklist for caregivers to take care of relatives at home that includes common items that caregivers may need to consider when providing care at home. It can help caregivers feel more prepared and confident in their role, and it can also make it easier for them to access the support and resources they need:

Maybe some checklist. What is common? Now your relative comes home, what is the most common thing you need to think about so that you can get help about what to do? A checklist, only bullet points might be helpful.

IC.13, Table 1

#### Information About Relevant External Domains

Most caregivers felt the need to have information about external domains, such as web pages with information, third-party applications for caregiving or mental health, and videos with exercises for caregivers. Some caregivers described that there were other support groups and information and resource portals for caregivers but not many knew about them. Therefore, it is important to obtain this information from the system:

There is a group here in XX [city name] that is only for relatives. And when I talk about it, not many people know about these groups. You as a relative can get there, talk, and participate in activities. Links to such groups where you might be reminded that I need support [as a relative]. I need support to be strong. I will not only be able to be strong by myself.

IC.13, Table 1

Most health care professionals stated that information was already available in these external domains and emphasized that this information should be presented in the web-based intervention. Some health care professionals pointed out that although there is information already present on the web, caregivers do not know where and how to access it:

I think that there is very good information from other resources, that such a portal could compile, that you provide a lot of links and contact information and, here you as a relative can get support from patient associations and relatives associations and so on, it is a jungle of information in itself, so the patient or relatives should have a place to go to where you can find very different kinds of information.

HCP.13, Table 2

#### Web-Based Forum

Among others, a web-based discussion forum emerged as an important preference for study participants to support caregivers’ well-being. Caregivers and health care professionals emphasized the importance of a web-based discussion forum in which stakeholders can share their ideas and experiences. Most caregivers felt that such a space would help them read about other caregivers’ situations and learn from their experiences. However, they had reservations about the credibility and accuracy of the information provided by the caregivers. Most caregivers in this study supported the idea of a web-based forum or peer-to-peer support. However, there are some barriers to using a web-based forum. For example, some caregivers use computers at work and do not want to continue using them at home. Some caregivers who have used social media excessively in their daily lives become tired of it and try to avoid any social media or similar activities. However, some caregivers also pointed out that although web-based forums can be useful, they can be generic while having a heterogeneous group of caregivers and hence, expressed a need to have a specific forum for caregivers of patients with HNC. Most caregivers and health care professionals also stressed the risk of spreading incorrect information in such forums during discussions with other caregivers, particularly those directly related to patient caregiving. To this end, health care professionals and caregivers highlighted the need for a moderator that would monitor the discussions and answer caregivers’ questions. Most caregivers in this study felt a sense of relatedness, acknowledgment, confirmation and engagement, and competence (based on positive design guidelines) through a web-based forum functionality.

#### Real-time Discussion and Chat With Other Informal Caregivers

Most caregivers highlighted their positive experiences with other web-based forums, where they could better understand their situation by reading about other caregivers’ situations. They also felt that sometimes these posts or texts included best practices that had worked for other caregivers, which they reported as being more trustable. In addition, real-time chats with other caregivers and health care professionals were highlighted as important features for caregivers. It would be helpful to have a real-time chat feature so that caregivers can use it to interact with health care professionals and other caregivers. A major advantage of this approach is its acknowledgment and relatedness. Caregivers should be able to share noncritical information and personal experiences in their daily lives. The caregivers felt that it was important to have a safe space to share their feelings and emotions. Sharing their experiences enables caregivers to obtain a sense of acknowledgment and confirmation. They feel that they are not alone, and there are more people in the same situation:

You learn a lot about how everyone feels, what symptoms they have, and what help they have received for the symptoms. It has been very good and very educational because you see others with the same disease.

IC.15, Table 1

Health care professionals also confirmed that web-based forums might help overcome social isolation. For example, if caregivers need information in the middle of the night when health care professionals are not available, a web-based forum asking for advice might be helpful. Some health care professionals also stated that if a caregiver is alone and has no friends or family available, they may get exhausted and may need support to overcome tiredness and loneliness. These conversations should be permanent on the forum so that health care professionals can check and comment on them later. This reduces the risk of spreading incorrect information:

You can be quite alone as a close relative. It also depends upon your family situation, if I talk about my patients, some of them have a quite large and active network of friends and family, and they all support the patients in different things. However, if you are the only person caring for the patient, you can be exhausted.

HCP.10, Table 2

#### Structured Format of Web-Based Forum

Most caregivers highlighted the need for different dedicated channels in web-based forums for various purposes. The forum could have different channels based on caregivers’ needs. For instance, a channel where caregivers can ask questions to health care professionals or a channel that is dedicated to questions related to the daily life problems of caregivers. They described that in daily life, they experienced problems that other caregivers might have encountered and might have some good solutions to. In this way, they could support each other by sharing their experiences and tips:

I would like to have a channel where I can really ask any type of question. It can be practical questions, such as: now I go on my knees, I need someone who can clean the home for me. Does anyone have any idea how I can handle this? Or they are doing construction work outside the house so it is not possible to stop with the car outside, how can I do to pick up my wife there.

IC.3, Table 1

Health care professionals stressed that they should answer questions related to the patient’s treatment, which should be permanently available to everyone. Health care professionals felt that this would ensure that questions were not repeated and that there was a repository of answers created together by caregivers and health care professionals that is available to all:

If you enter the questions and the healthcare professionals may answer the questions and the answer should be available for everyone in the forum, so if you post a question everyone can see the question the most suitable person can answer. The answers should also be permanently available so that the others can get benefit from them.

HCP.9, Table 2

### Virtual Meeting Place

Virtual meeting place emerged as another important function for the web-based intervention. Caregivers would like a place where they can talk to each other and share their experiences and knowledge by being present on a video call. These video meetings can also be used to interact with and receive advice from health care professionals. For example, if caregivers need to discuss their situation with a social worker (curator in a hospital), they should be able to do so using this feature. They suggested that web-based meetings could benefit caregivers for socialization and trust building with other people. Some caregivers provided positive and emotional reflections on the virtual video discussion sessions with other caregivers. They also suggested that such video meetings and discussions might be good for social interaction, engagement, and trust building (based on positive design guidelines) among caregivers with similar circumstances. However, most caregivers highlighted the barriers to these video activities. These web-based video meetings and events are good for easy accessibility, but caregivers might miss human contact in real-life meetings. Some caregivers preferred to meet each other in real life, although they agreed on meeting on the web when they cannot meet in real life. There are also technical issues with these web-based video meetings, and many caregivers and health care professionals did not have good experiences. They were concerned about facing the same problems for web-based video meetings during this intervention:

I did not know what this meeting would mean to the others. But a reflection from my side is that God, how nice it has been to meet you all...tears have come out of my eyes, I tried not to burst out completely in tears, it has been very, very nice to share emotions. And as someone said here that we did not know each other before, but still pretty quickly you get a connection with each other, and it is very liberating. Very, very nice so thank you very much, everyone.

IC.13, Table 1

Health care professionals also emphasized that there should be a place where caregivers can discuss their problems and feelings with other caregivers in similar situations. They felt that caregivers might not share their feelings with their relatives because of an underlying sense of guilt. In this situation, there should be an outlet for sharing feelings and emotions:

Yes, I think you should discuss what you think. If you have such a support function where you could step in as a relative e.g. God, I think this is tough with my husband, he smells bad, we in the family can’t eat what we want, but I don’t want to say it outright because it’s my husband who has this situation, it’s not me who should feel pity, although it will be a pity for me, still you should express those feelings, there should be a forum for me to be able to talk about all this.

HCP.2, Table 2

### Chatbot

The chatbot was the least desired function by both the caregivers and health care professionals. Only a few caregivers felt positive about it being a good and easy way to access information. However, these caregivers were proficient in using this technology. They felt that they could trust the information from a chatbot, as it works similarly to a search engine, such as Google. According to many caregivers, one of the vital barriers to adopting chatbots was the lack of human contact. They emphasized the need to interact with a person when asking for critical health-related information. Although many acknowledged the relevance of chatbots for general information retrieval, they preferred to communicate with people either verbally or in written form. The lack of trust in robotic technologies has also emerged as a potential barrier to adopting chatbots. Most participants were concerned about the credibility of the information obtained from the chatbots. They were hesitant to retrieve critical information related to their relative’s health from chatbots. However, the limited knowledge of the participants regarding chatbots was also seen as a reason for this distrust. Some participants explained that they need to gain a better understanding of the basic mechanisms of chatbots before they could consider using them. Some caregivers who had previously used chatbots felt that they needed more specific answers as opposed to generic responses that they thought chatbots provided. They felt annoyed by the generic answers to their contextual and specific questions:

I’m a little hesitant to talk to an artificially intelligent robot in these fragile circumstances. I feel it is not the same as a person with flesh and blood that I have in front of me. So, I’m hesitant there...So, the first thought that comes to the mind when something happens, is you want to talk with someone you know well. Is there anyone who can help me, who can talk, and I think, usually you get much calmer if you talk to a real person rather than a robot...

IC.10, Table 1

Similar to caregivers, health care professionals have also suggested the use of chatbots for basic and noncritical information retrieval. For example, tips and suggestions for preparing food for patients might be a good use of such chatbots. Therefore, chatbots might be good for basic information, for example, as nutrition tips. However, they were generally hesitant to interact with robots. Health care professionals have suggested a combination of humans and robots to provide information. Both should complement each other by providing different types of information:

I think it can be both like with real people responding to some things and an AI robot responding to some other things. One does not have to exclude the other, you can have both. And both should complement each other and then they can also seek support from each other.

HCP.9, Table 2

A summary of the preferred functions, desired characteristics, and facilitators and barriers is presented in Table 3.

**Table 3:** Carer eSupport’s preferred functions, characteristics, and the facilitators and barriers.

Functions	Desired characteristics	Facilitators	Barriers
Information	Information about different diagnoses and treatments with easy-to-understand and nonmedical language Psychological help for caregivers’ well-being Checklist of daily life activities with the patient Digitalization of paper-and-pen–based information Links to already available information from external resources Links to other groups and portals for the relatives of the patients Inspirational and motivational videos	Tailor-made and trustable information Collected information in one place Help for caregiving the patient Help in daily life activities Help for caregivers’ well-being and mental health Enhances positive emotions such as virtue, acknowledgment, confirmation, and competence	Much information is already available and provided by health care professionals Very general information
Web-based forum	The forum should be moderated by the health care professionals Real-time discussion and chat with caregivers Possibility to send private messages to the forum members Permanently available conversations Dedicated channels for different types of discussions, for example, daily life problems of the caregivers, patients’ health-related issues, caregivers’ health and physiological well-being, and asking questions about head and neck cancer treatments	Easy availability and accessibility of information Helpful to combat loneliness, depression, and anxiety Share personal feelings Enhances positive emotions such as relatedness, acknowledgment, confirmation, engagement, and competence	Risk to spread wrong information Availability of other web-based forums and groups Antisocial media people
Virtual meeting place	Possibility for video meetings with the health care professionals Real-time chat and discussions with health care professionals Possibilities for seminars and group discussions Possibility for video meetings with health care professionals	Enhances positive emotions such as social trust, relatedness, acknowledgment, confirmation, engagement, and competence	Technical issues Human contact is missing
Chatbots	A combination of human and robot Only for noncritical information	Easy availability and accessibility of information	Human contact is missing Lack of trust in robots Lack of knowledge about chatbots Standard answers

## Discussion

### Principal Findings

This study explored HNC caregivers’ main preferences and desired functionalities for Carer eSupport. These needs of caregivers were also explored and discussed with highly experienced and qualified health care professionals in different areas of HNC treatment. Our main focus was on understanding caregivers’ situation and highlighting their preferred functions that may enhance their subjective well-being. Through focus groups with caregivers and health care professionals, we discussed 4 major functions: information, a web-based forum, a virtual meeting place, and a chatbot. The detailed characteristics and content of these functions were also discussed with the study participants according to the caregivers’ situation and context. Moreover, we highlighted the facilitators and barriers to the successful implementation of Carer eSupport. The potential barriers also guided the study participants to discuss the characteristics of their preferred functions, which might help them avoid those barriers. We now discuss our findings with previous studies on users’ needs, well-being, and adoption of eHealth applications.

Previous literature has highlighted tailored information and peer-to-peer support as the primary needs of caregivers [7,8,27,28]. This study extends this knowledge by providing functions to address those needs and an in-depth understanding of the special characteristics and content followed by facilitators and barriers to these functions. Jansma et al [27] stressed that caregivers of patients with palliative cancer need support to communicate better with health care professionals and other caregivers, which is in line with our findings for caregivers of patients with HNC. Köhle et al [7] suggested that peer-to-peer support and information for caregiving are the most important needs of caregivers; however, their study focused only on the partners of patients with cancer and their psychological well-being. The above-described studies give us an idea of caregivers’ needs in different contexts; however, they did not suggest how to address the need for a web-based intervention that our study addresses in the form of different functions, such as a web-based forum and virtual meeting place for peer-to-peer support and communication with health care professionals.

This study also provides insights into the context of caregivers when designing a web-based intervention that might support their subjective well-being. Many studies have focused on the needs and preferences of patients for technology-enhanced systems [29-31]; however, caregivers seem to be a neglected user group in eHealth research [32]. To the best of our knowledge, this is the first study to discuss the preferences of caregivers of patients with HNC and the potential facilitators and barriers to the successful implementation of this web-based intervention focusing on caregivers’ well-being. The inclusion of caregivers and health care professionals in this study is also a distinct feature not commonly seen in previous research, which provides additional insights.

Previous studies on caregivers of patients with cancer have also suggested the need for a wide range of tailored information [7,8,27,28]. Caregivers want to be more aware of the patient’s medical condition but lack information about existing support [7]. Our results also highlighted that caregivers need information about the different impairments caused by HNC, their treatments, and how they (both patients and caregivers) can be prepared for those treatments. After treatment, they also wanted to know the long-term side effects and the recovery process. Caregivers also want a checklist for daily life activities that they need to perform with patients. The checklist includes common items that caregivers may need to consider when their relatives come home. This can help caregivers feel more prepared and confident in their role, which can in turn improve the quality of care that patients receive at home [28].

Previous research has shown that living with severely impaired patients may create depression and anxiety in caregivers, which might affect caregivers’ psychological health and well-being [6,28]. In our study, to cope with the problems related to psychological health, both health care professionals and caregivers highlighted the importance of informative and inspirational material. It can be helpful for caregivers to hear about the experiences of others who have faced similar challenges while caring for loved ones with cancer. Inspirational videos featuring stories from other caregivers can provide support and encouragement, as they offer a glimpse of the struggles of others who have experienced similar situations. Previous research on design for well-being suggests basic elements that may enhance users’ psychological well-being by addressing their needs [15,33]. In our study, we found that by providing tailor-made and trustable information, some of the needs for psychological well-being can be addressed, namely, virtue, acknowledgment, and competence.

Our findings highlighted the importance of a web-based forum for caregivers to communicate and share their views with other caregivers and ask questions to health care professionals. In some cases, caregivers experience loneliness, depression, and anxiety, and they believe that connecting and interacting with other caregivers can be helpful [34]. In addition, recent studies have also indicated that feeling connected with a community might reduce the loneliness and social isolation of relatives of patients [34,35]. Previous research on patients with cancer and their relatives has shown the positive effects of peer support and connecting people [36,37]. Köhle et al [7] stressed the need for peer support to overcome depression and anxiety among partners of patients with cancer. They suggested a peer support function in a web-based intervention for patients’ partners to support their psychological well-being and to provide them with the acknowledgment of what they are going through in their lives and struggles. In our findings, caregivers also wanted to communicate with each other to acknowledge and confirm their daily life activities with the patients.

It is evident from previous research on designing for well-being that connecting and socializing with other people in the same situation give a sense of happiness and motivation and enhance positive emotions [15,16,38]. Peters et al [17] also highlighted “Relatedness (being connected to other related people)” as a basic element for the psychological well-being of people. Studies have highlighted that informal caregivers tend to search for relevant web-based forums and communities to feel a sense of relatedness and social belonging through them [35]. In this study, we found that a web-based forum may address the needs of caregivers’ subjective well-being by providing relatedness, acknowledgment, confirmation, and engagement. Our findings on the need and effectiveness of a web-based forum are in line with previous research; however, we provide detailed characteristics and content of the web-based forum so that the facilitators of technology adoption can be enabled and barriers can be minimized. For instance, the risk of spreading wrong information is huge in the web-based forum; to overcome this issue, our findings suggest that health care professionals should moderate the forum, and the communication between the users should be permanently available so the moderator may monitor the communication and make corrections if needed.

Our findings suggest a virtual meeting place in which caregivers can meet on the web and share their feelings, experiences, and knowledge. The caregivers described that web-based forums were good for written communication, but web-based meetings and sessions with health care professionals and other caregivers provided better interaction. The positive effects of the web-based forum and the virtual meeting place are the same; however, virtual meetings provide some additional benefits such as trust building, knowing each other better, and emotional engagement with others who are in the same situation. Therefore, in virtual meetings, the components of well-being are the same as those suggested in the web-based forum, with social trust as an additional component. The caregivers suggested that web-based meetings or seminars should be conducted with other caregivers and health care professionals. The basic idea here is to provide caregivers with peer support and valuable information from health care professionals about caregiving to the patients and taking care of their own well-being.

Our findings also suggest that virtual meetings cannot be a complete substitute for real-life meetings, but they should complement real-life meetings. Caregivers considered virtual meetings good because of their easy accessibility, but they also met people in real life and miss human contact. Recent studies have shown that virtual meetings may positively influence users’ well-being [39], such as engagement and motivation; however, people still prefer physical meetings [40]. Hence, virtual meetings complement traditional physical meetings and should be used only when real-life meetings are impossible or feasible. As most Swedish populations are dispersed throughout the country, virtual meetings may be a good alternative to real-life meetings.

Previous research on patients with cancer and their caregivers highlights the importance of peer support in the informal caregiving context [7,36,37]; however, the questions of how this support should be given to caregivers and which medium should be used have not been explicitly answered from the end users’ perspective. In this study, we discussed the different methods and mediums for providing peer support such as the web-based forum and virtual meeting place; both provide peer support and connect caregivers with their facilitators and barriers.

Generally, our study participants did not like the idea of a chatbot asking questions and retrieving information. Caregivers wanted human contact while communicating their concerns and feelings. Previous studies on the role of chatbots in cancer caregiving also suggest that human elements are important and cannot be replaced by robotics [41,42]. In our study, the lack of trust in robotics technologies was another barrier to chatbot adoption. In particular, caregivers who did not know how chatbots worked were reluctant to use them. The trust issues on chatbots are also evident in previous research on chatbots for cancer caregiving [41-43]. People doubt the credibility and accuracy of the information received from chatbots. The lack of knowledge about how artificial intelligence and machine learning work behind retrieving information and data also create distrust in chatbots [43]. Therefore, we suggest proper education and training for users to adopt chatbots successfully in their daily lives.

The study participants suggested that the chatbot should cooperate with health care professionals rather than exclude them from each other. Caregivers and health care professionals have suggested that chatbots can be used for general and noncritical information. However, health care professionals should directly ask questions regarding medical treatment and patient health. Previous research also suggests that chatbots have the potential to integrate into the health care system, but they should not replace health practitioners, and both might work side by side [42,44].

### Positive Design Framework for Informal Caregivers’ Well-being

A general understanding of overall human well-being might be helpful for HCI researchers and designers when designing eHealth interventions; however, user interactions and experiences are usually contextual and can vary based on their circumstances and dynamics [45]. The user context also plays a vital role in the successful adoption of eHealth interventions. Designers should involve users in the design process and deliberately focus on their well-being and positive emotions. Hence, the user-centered design approach is of utmost importance for caregivers’ well-being; after all, they are the true evaluators of their well-being [4].

In this study, we incorporated general guidelines of designing for well-being and positive design in the context of caregivers of patients with HNC. We drew from 3 studies (as presented in the introduction section) that used positive psychology principles to propose general guidelines and frameworks for well-being design [15-17]. These studies highlighted 8 components of human psychological needs that should be considered when designing for well-being: virtue, personal significance, pleasure, autonomy, competence, relatedness, achievements, and engagement. Empirical evidence from our study and previous related studies indicates that not all 8 components of designing for well-being are applicable in the caregivers’ context; personal significance and pleasure were found to be not applicable to caregivers of patients with HNC. Instead, we explored 3 other components not explicitly proposed in previous studies but relevant to caregivers’ contexts: acknowledgment, confirmation, and social trust. These components emerged from the focus groups.

**Figure 2:**
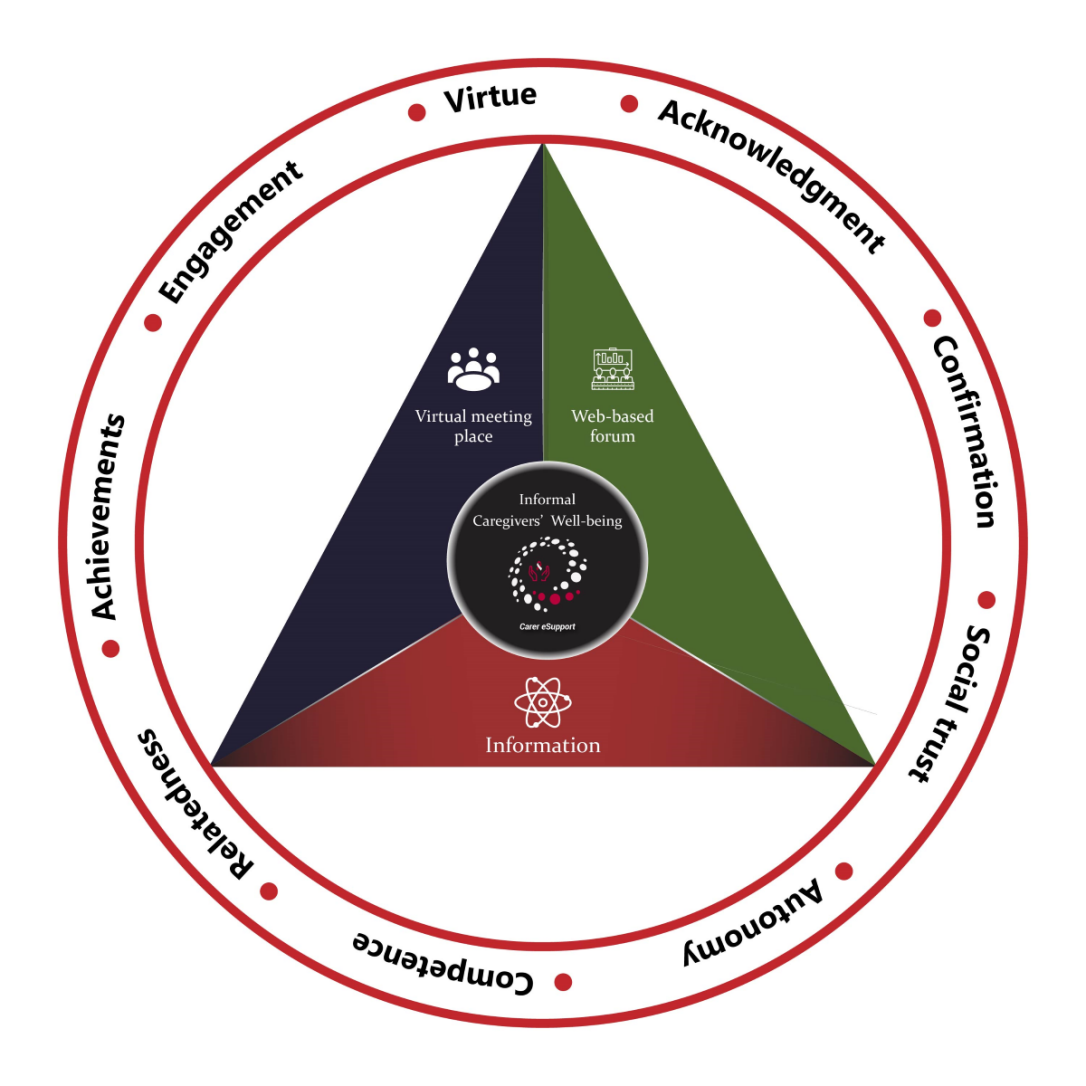
Positive design framework for informal caregivers.

In Figure 2, we present the positive design framework to support caregivers’ well-being. In total, 3 major preferences of caregivers emerged as the main functions of Carer eSupport: information, web-based forum, and virtual meeting place. Each function independently stimulates caregivers’ well-being, and the intersection of these functions is where caregivers may feel supported and flourish. The outer circle presents the basic components of caregivers’ well-being that these 3 functions can achieve: virtue, acknowledgment, confirmation, social trust, autonomy, competence, relatedness, achievements, and engagement. Each function may independently address many components, and each component can be achieved by more than one function (also indicated in the *Results* section). For example, acknowledgment and confirmation can be achieved in all 3 functions: information, web-based forum, and virtual meeting place. Similarly, relatedness can be achieved with 2 functions: web-based forum and virtual meeting place. However, social trust or trust in other people is easier to achieve in a virtual meeting place.

### Limitations

In this study, we introduced a positive design framework to support caregivers’ well-being. A limitation of our study is that it was performed with a rather small group of caregivers. To explore the detailed implications of the proposed framework, more empirical evidence with a larger group of caregivers is needed. Another limitation is that the framework was not tested for usability and user experience (UX); therefore, we might not have established the framework’s effectiveness from a UX perspective. However, in future research, we will test the usability and feasibility of Carer eSupport and the proposed framework.

This study was undertaken during the pandemic (COVID-19), and all focus groups were conducted on the web to ensure the study participants’ health. Research on the ethical aspects of qualitative studies shows that the well-being and overall health of study participants should always be prioritized over research objectives and strict timelines [46]. Therefore, we could not use the full potential of focus groups in real-life settings. The use of a nonprofessional translator to translate the data collected in the focus groups conducted in the Swedish language could be another potential limitation. There is a risk that translations may not fully capture the nuances of the original language, which could affect the study’s overall results.

### Conclusions

The design strategies for caregivers’ subjective well-being, especially the caregivers of patients with severe diseases such as cancer, have been scarce in previous HCI research. This study revealed an in-depth understanding of caregivers’ contexts and their preferred functions for a web-based intervention (Carer eSupport). Health care professionals’ valuable input on the preferred functions gave us important insights into the adoption and practical implementation of Carer eSupport in routine cancer care. Our empirical findings on the potential facilitators and barriers to adopting Carer eSupport allowed us to define the special characteristics of caregivers’ preferred functions so that facilitators can be included and barriers can be omitted in the intervention. We used the theoretical foundation of designing for well-being and positive design in the informal caregiving context and proposed a positive design framework to support informal caregivers’ well-being. Our proposed framework might be helpful for HCI and UX researchers to design meaningful eHealth interventions with a clear focus on users’ well-being and positive emotions, especially for caregivers of patients with HNC. The importance of involving users in the design and development process to solve objective problems has been demonstrated in previous research. However, the HCI research community should focus more on holistic approaches and the subjective well-being of end users.

## Abbreviations

HCI: human-computer interaction
HNC: head and neck cancer
RQ: research question
UX: user experience
